# Mesenchymal Stem Cells Improve Rheumatoid Arthritis Progression by Controlling Memory T Cell Response

**DOI:** 10.3389/fimmu.2019.00798

**Published:** 2019-04-16

**Authors:** Noymar Luque-Campos, Rafael A. Contreras-López, María Jose Paredes-Martínez, Maria Jose Torres, Sarah Bahraoui, Mingxing Wei, Francisco Espinoza, Farida Djouad, Roberto Javier Elizondo-Vega, Patricia Luz-Crawford

**Affiliations:** ^1^Laboratorio de Inmunología Celular y Molecular, Centro de Investigación Biomédica, Facultad de Medicina, Universidad de los Andes, Santiago, Chile; ^2^Escuela de Ingeniería Bioquímica, Pontificia Universidad Católica de Valparaíso, Valparaíso, Chile; ^3^IRMB, INSERM, Univ Montpellier, Montpellier, France; ^4^Cellvax, SAS, Parc BIOCITECH, Romainville, France; ^5^Cells for Cells, Universidad de los Andes, Santiago, Chile; ^6^Laboratorio de Biología Celular, Departamento de Biología Celular, Facultad de Ciencias Biológicas, Universidad de Concepción, Concepción, Chile

**Keywords:** mesenchymal stem cells, rheumatoid arthritis, T cell, plasticity, immunomodulatory

## Abstract

In the last years, mesenchymal stem cell (MSC)-based therapies have become an interesting therapeutic opportunity for the treatment of rheumatoid arthritis (RA) due to their capacity to potently modulate the immune response. RA is a chronic autoimmune inflammatory disorder with an incompletely understood etiology. However, it has been well described that peripheral tolerance defects and the subsequent abnormal infiltration and activation of diverse immune cells into the synovial membrane, are critical for RA development and progression. Moreover, the imbalance between the immune response of pro-inflammatory and anti-inflammatory cells, in particular between memory Th17 and memory regulatory T cells (Treg), respectively, is well admitted to be associated to RA immunopathogenesis. In this context, MSCs, which are able to alter the frequency and function of memory lymphocytes including Th17, follicular helper T (Tfh) cells and gamma delta (γδ) T cells while promoting Treg cell generation, have been proposed as a candidate of choice for RA cell therapy. Indeed, given the plasticity of memory CD4^+^ T cells, it is reasonable to think that MSCs will restore the balance between pro-inflammatory and anti-inflammatory memory T cells populations deregulated in RA leading to prompt their therapeutic function. In the present review, we will discuss the role of memory T cells implicated in RA pathogenesis and the beneficial effects exerted by MSCs on the phenotype and functions of these immune cells abnormally regulated in RA and how this regulation could impact RA progression.

## Introduction

Mesenchymal stem cells (MSCs) are multipotent stem cells able to exert immunosuppressive functions on both the innate and the adaptive immune cells (1). They have been isolated from almost all mesodermal tissues including bone marrow, adipose tissue, umbilical cord blood, umbilical cord, placenta, menstrual fluid, and dental pulp (2–5). The International Society for Cellular Therapy (ISCT) has defined minimal criteria for characterizing MSCs that include a fibroblastic-like morphology, the expression of mesodermal markers such as CD90, CD105, and CD73, the lack of hematopoietic marker expression such as CD45, CD34, CD14, and the capacity to differentiate into adipocytes, chondrocytes and osteoblasts (6). MSCs have been reported as an interesting therapeutic cell candidate for the treatment of autoimmune diseases such as RA, due to their capacity to attenuate the exacerbated pathogenic immune response observed in these patients (7). However, given the complexity of RA disease as well as the mechanisms involved in MSC immunosuppressive functions, it is mandatory to decipher the mechanism by which MSC mediated their immunosuppressive potential on the immune cell subsets associated to RA to improve MSC-based therapy. In this context, one of the main target for MSCs-based therapy are the pathogenic memory T cells due to their critical role in autoimmune disease progression including RA (8). Currently there is no article focusing in discussing the importance of targeting-memory T cells with MSCs-based therapy for autoimmune disease treatment.

Therefore, in this review, we will focus on the effect of MSCs on memory CD4^+^ T cells subsets and we will discuss about the advantage that this knowledge could render to improve their immunosuppressive properties in order to develop novel MSCs-based therapy for RA treatment. During the development of this review, we will discuss about the role of memory T cells in the evolution of autoimmune disease focusing on RA and we will infer studies between MSCs and their impact in memory T cells and how the regulation of this populations could be a key player on RA improvement.

## MSC-Based Therapy for Autoimmune Disease Treatment

MSCs have been largely propose as a therapeutic tool for autoimmune disease treatment due to their potent suppressive activity to inhibit proinflammatory cells from both the innate and adaptive immune system. Indeed, it has been reported that MSCs are able to modulate the differentiation and function of myeloid cells toward immunosuppressive phenotypes. These cells includes monocytes (9, 10), dendritic cells (DCs) (11, 12), macrophages (13), myeloid-derived suppressor cells (MDSCs) (14), and neutrophils (15). Furthermore, MSCs inhibits the proliferation of T cells (16, 17) and B cells (18), as well as their functions. The mechanisms involved in this immunomodulation include cell-cell contacts and the production of soluble factors (19). Besides, MSCs are able to migrate to inflammatory sites in order to interact and modulate proinflammatory immune cells in the site of inflammation (20). For all this reasons, we can currently count a totally of 707 MSC-related clinical trials registered on the NIH Clinical Trial Database (https://clinicaltrials.gov/). These clinical trials mainly tend to evaluate the therapeutic efficacy and safety of MSCs from different sources. Moreover, until December 2018 exists several clinical trials targeting autoimmune disease treatment such as Multiple Sclerosis (MS) (*n* = 29), Crohn's Disease (*n* = 7), systemic lupus erythematous (SLE) (*n* = 12), and RA (*n* = 14). In general, the short-term and long term use of MSCs based therapy give positive effects with no report of serious adverse events besides some immediate type I hypersensitivity (pruritis, rash, fever) in <15% of patients (21). For example, Riordan et al. evaluated the safety and efficacy of the intravenous administration of umbilical cord-derived MSCs (UC-MSCs) for the treatment 20 MS patients (22). MS is an inflammatory disorder of the brain and spinal cord in which focal lymphocytic infiltration leads to damage of myelin and axon (23). The authors demonstrated that after 1 year, MRI scans of the brain and the cervical spinal cord showed inactive lesions in 83.3% of the subjects followed (22). In another study, an allogeneic adipose-derived stem cells (ASCs) was used in a phase I/IIa clinical study for Crohn's disease treatment (24). Crohn's disease is a systemic inflammatory chronic disorder that affect the digestive tract (25). ASCs based treatment showed that 69.2% of all the patients had a reduction of the number of draining fistulas after 24 weeks post-injection compared to the placebo group. Moreover, this study demonstrated that eASCs infusion was safe and a beneficial therapy to treat perianal fistula of Crohn's disease patients (24). Finally optimistic results have been obtained for SLE treatment using MSCs (26). SLE is a multisystem autoimmune disease characterized by inflammation of multiple organs owing to in part by loss of tolerance to self-antigens and the production of autoantibodies (27). Wang et al. demonstrated that after 12 months using two intravenous infusions of UC-MSCs in 40 patients with refractory SLE a well-tolerated safety profile with 32.5% (13/40) of patients achieving a major clinical response and a significant decrease in disease-activity (26).

However, despite these results there are still a lot of controversy regarding the positive effects of MSCs based therapy since their effect strongly depends on the etiology of the disease and the degree of inflammation. Thus, it is very important to understand the interaction between MSCs and pathogenic immune cells such as memory T cells since they are main players in the generation, pathogenesis, and progression of autoimmune disease.

## Memory T Cells: Key Player in the Pathogenesis of Autoimmune Disease

After infection or immunization, naive T cells undergo a clonal expansion leading to a high frequency of antigen-specific T cells with a rapid effector function. Naïve CD4^+^ T cells can differentiate into multiple effector T helper (Th) cell subsets such as Th1, Th2, Th17, and T follicular helper (Tfh) cells among others, while naïve CD8^+^ T cells differentiate into cytotoxic T lymphocytes (CTLs) (28). Once the initial response of the adaptive immune system against an antigen ends, the organism must return to the homeostasis through the contraction of effector T cells. During this period the small amount of cells that survive will eventually become part of the immunological memory: immune cells that are able to respond rapidly to a second round of a specific antigen previously encountered (29). The generation and persistence of memory T cells is an important feature of the adaptive immune system acquired following antigen exposure that provides lifelong protection against infections (30).

Memory T cells are an heterogeneous population of cells classically distinguished by the expression of the CD45RO isoform and by the absence of the CD45RA (CD45RO^+^CD45RA^−^) (31, 32). Lately, in human, specific subsets of memory CD4^+^ and CD8^+^ T cells in peripheral blood mononuclear cells (PBMCs) were identified through the expression of CC-chemokine receptor 7 (CCR7), a chemokine receptor that controls the homing to secondary lymphoid organs (33). CCR7 negative memory T cells were found to produce more effector cytokines, compared to the CCR7 positive subset (34). Based on this finding, two subsets of memory T cells were identified: CCR7^+^ central memory T cells (T_CM_) and CCR7^−^ effector memory T cells (T_EM_) (33). Several studies have been carried out to characterize the memory cells present in PBMC using an extensive panel of markers. The CD44^hi^, CD45RO^hi^, CD45RA^low^, CD127^hi^, CD62L^hi^CCR7^hi^ T_CM_ cells are generated and reside in secondary lymphoid tissues in the absence of antigen while CD44^hi^, CD45RO^hi^, CD45RA^low^, CD127^hi^, L-selectin^low^ CCR7^low^ T_EM_ cells, are generated in secondary lymphoid tissues and recirculate between blood and non-lymphoid tissues in the absence of antigen (33).

As mentioned before, the long-lived memory T cells in the presence of secondary antigen exposure expand and develop a more robust and stronger response. In the case of autoimmune diseases memory T cells might become harmful against self-antigens since these memory cells exhibit a potent pathogenic response against self-tissues. Moreover, due to their longevity, they are very difficult to eliminate thus the development of novel therapies directed against these cells are of main importance to control autoimmunity.

In this context, the role of memory T cells in autoimmune diseases has been studied. MS patients have an elevated numbers of memory T cells (35–37), particularly of the T_EM_ subsets (38, 39). Recently it has been reported that memory CD4^+^ CCR9^+^ T cells are altered in MS patients and they could be mediate the development of secondary progressive MS progression (40). Also, it has been reported that memory T cells subpopulation are increased in active Crohn's disease patients (41, 42). Indeed, peripheral blood and intestinal mucosa memory T cells from active Crohn's disease patient have an increased intracellular production of TNFα and correlate with the score of the disease (CDAI). In addition, this peripheral blood memory T cells-producing TNFα have an increased migratory profile to extra nodal lymphoid tissues such as the intestinal mucosa (43). Furthermore, there is evidence suggesting an augmentation of CD4^+^ T_EM_ cells population in SLE pathogenesis (44). Also, the PD1^+^ICOS^+^T_CM_, and PD1^+^ICOS^+^T_EM_ subpopulation are increased in SLE patients and T_EM_ positively cells correlated with the severity of the disease (45). Likewise, it has been observed an enrichment of CD4^+^ T_EM_-cell associated genes within SLE loci, Crohn's loci and RA loci (46). All this evidence point memory T cell subsets as major contributors of autoimmune pathogenicity.

### Role of Memory T Cells in the Development and Progression of RA

RA is an autoimmune disease characterized by the high production of auto-antibodies affecting a wide variety of auto-antigens. Among them, the rheumatoid factor (RF) and anti-citrullinated protein antibodies (ACPAs) have been the most described (47). RA immunopathogenesis is characterized by deficiencies in the immune response with predominance of pro-inflammatory cells and an alteration of the peripheral immune tolerance which involves in particular CD4^+^ T cells (48, 49). CD4^+^ T cells of RA patients undergo a premature transition from a naïve to a memory phenotype. The resulting memory CD4^+^ T cells are hyper-proliferative because of failures in the cell cycle checkpoint which promote their differentiation toward Th1 and Th17 pathogenic T cells (50). This was confirmed in studies demonstrating that RA patients have large numbers of memory CD4 T cells that infiltrate the inflamed synovial membrane (51–55). Moreover, the increased frequency of T_EM_ cell subset was observed in the synovial fluid from RA patients (55). While T_EM_ cells have a short lifetime they possess a potent effector function with a high capacity to secrete pro-inflammatory cytokines allowing them to respond faster to antigens present in the synovial fluid (34). All together, these studies suggest the presence of highly activated and differentiated memory CD4^+^ T cells with a high capacity to produce pro-inflammatory cytokines in synovial fluid of RA patients.

### Conventional Therapy for RA Treatment

A large variety of drugs aiming at reducing the symptoms and gradual progression of the disease are currently available. Among them, synthetic disease-modifying anti-rheumatic drugs (sDMARDs) including methotrexate (MTX), leflunomide, sulfasalazine, and hydroxychloroquine, biologic response modifiers referred as biologics (bDMARDs) and corticosteroids. All these treatments target inflammation and are aimed at improving both the quality of life and prognosis of RA patients (56) through the prevention of structural damage (erosive disease) and control of extra-articular symptoms. Since, RA pathogenesis is associated to alterations of immune cell functions and cytokine secretion produced in part by pro-inflammatory CD4^+^ T memory responder cells, a wide variety of bDMARDs have been proposed to target the latter cells. For instance, the first bDMARD tested was aimed at reducing the production of tumor necrosis factor alpha (TNF-α) (Infliximab), a pro-inflammatory cytokine highly produced by memory T cells of RA patients (57). Since then, other TNF-targeting agents such as etanercept, adalimumab, certolizumab, and golimumab as well as other biological agents such as anti-IL6 (tocilizumab), anti-CTLA4 (abatacept), and anti-CD20 (Rituximab) were developed (56). However, the treatment of some RA patients with TNF inhibitors did not significantly reduce the frequency of pathogenic Th17 cells revealing that a high range of patients do not respond to this treatment (57). Later, an anti-interleukin 17 (IL-17) antibody (secukinumab) and anti-IL-17RA antibody brodalumab (AMG827) were developed and evaluated in clinical trials including RA patients with an inadequate response to methotrexate. The phase II clinical study on RA patients demonstrated that the administration of brodalumab did not improve RA progression as revealed by the minimal response criteria set designed by the American College of Rheumatology (ACR) (58). Similar results were observed after secukinumab administration in a phase Ib clinical study that included moderate to severe RA patients (59). Indeed, the administration of these drugs did not reduce the frequency of memory Th17 cells. Interestingly, patients with RA treated with TNF inhibitors, possess pathogenic Th17 cells with a deleterious phenotype because of the high production of granulocyte-macrophage colony-stimulating factor (GM-CSF) (57). Indeed, GM-CSF is indispensable for the differentiation of inflammatory dendritic cells (infDCs) inducing the activation of memory CD4^+^ T cells producing IL-17 (60, 61). Thus, a monoclonal antibody against GM-CSF has been developed and described to be effective in clinical trial for RA treatment (62). However, despite this promising result, the use of the anti-GM-CSF antibody has not yet been approved (62).

Inhibitors of the Janus kinases (JAKs), such as Tofacitinib and Baricitinib, have also been developed for RA treatment (63, 64). These inhibitors block the activation of signal transducer and activator of transcription (STATs) signaling pathways, which drive the signature of many cytokines including interleukin-7 (IL-7) and interleukin-15 (IL-15) that are important for memory T cells proliferation and survival (64–66). Another approach was the development of drugs that mimic mechanisms naturally produced by our own immune system. For example, Abatacept is a soluble recombinant human fusion protein comprising the extracellular domain of human cytotoxic T-Lymphocyte Antigen 4 (CTLA-4). This protein binds to CD80 and CD86 receptors on the antigen-presenting cells (APCs) and blocks the interaction with T cells through the co-stimulatory molecule CD28 (67). Clinical trials have shown promising results using Abatacept for RA treatment (68). However, a subset of tissue-infiltrating CD4^+^ T cells from a group of RA patients have been shown to lose the expression of CD28 while starting to express memory markers (54, 69). These latter cells exhibit a high capacity to produce pro-inflammatory cytokines such as interferon-gamma (IFNγ) and TNFα and cytotoxic activity (69–73). Remarkably, the effect of bDMARD administration on memory T cell population has never been addressed.

Although a significant progress has been made with the current state of the art RA treatment for obtaining long-term remission-induction, still between 20 and 30% of patients with moderate-to-severe RA do not positively respond to mono or combinations therapy (plus Methotrexate) with these agents (74) thus the development of novel therapies targeting pathogenic memory T cells seems to be ideal to improve RA progression.

### MSC-Based Therapy for RA Treatment

Despite the fact that MSCs based therapy for RA treatment is one of the main autoimmune disease model use to study the mechanism underlying the therapeutic effect of MSCs, nowadays, RA MSCs-based clinical trials has been the least studied within the autoimmune diseases. In this context, exist 14 MSC-based therapy clinical trials for RA. Upon them, it has been reported that the intravenous infusion of allogeneic bone marrow and umbilical cord-derived MSC in a small group of refractory RA patients resistant to the anti-TNF monoclonal antibody therapy, led to a reduced erythrocyte sedimentation rate, improvement on DAS28 clinical score and diminished on the serum anti-cyclic citrullinated peptide (anti-CCP) antibody level, indicating the efficacy of MSC treatment. However, the observed clinical improvement was only partial and temporary because of the short term follow-up (75). In another study, using allogeneic UC-MSCs for RA treatment, the safety and effectiveness was demonstrated in a larger number of patients (76). In this study, MSCs and DMARDs were co-administrated intravenously in 172 patients with active RA inducing a significant increase in the percentage of regulatory CD4^+^ T cells (Treg) in the blood together with a significant clinical improvement for up to 6 months. Moreover, repeated infusion of MSCs after this period allowed an increased therapeutic efficacy of the cells (76). More recently, in a phase Ib/IIa clinical trial, the intravenous administration of allogeneic expanded adipose-derived stem cells (ASCs) in a study that included 53 patients with a placebo group was shown to be safe and well tolerated in refractory RA patients (77).

Unfortunately at today there is no report that shows an immune-monitoring of RA patients after MSCs infusion that could allow us to compare the immune profile of RA patients treated or not with MSCs with their clinical score before and after MSCs infusion. Indeed, it is mandatory to deepen on how MSCs affect the proinflammatory cells that are deregulated in these patients in particular pathogenic memory T cells. This information will surely help us to understand the mechanism by which MSCs exert their therapeutic function that will allows us to improve MSCs-based therapy.

## Immunomodulatory Role of MSCs on Memory T cells: Focus on RA

Despite the significant advances that have been made in the generation of novel therapies against RA, there are still a lot of patients that do not respond to any treatments. Hence it is reasonable to think that the resistance of pathogenic memory T cells could be the main contributor to the absence of a beneficial effect of these immunomodulatory therapies (78, 79). Therefore, it is mandatory for the successfully development of RA therapies to target these specific T cells subsets. In this context, the effect of MSCs on memory T cells have been investigated. For example, Pianta et al. demonstrated that the conditioned medium derived from the mesenchymal layer of the human amniotic membrane (CM-hAMSC) strongly inhibits central memory (CD45RO^+^ CD62L^+^) as well as effector memory (CD45RO^+^ CD62L^−^) T cell subsets, although the later ones to a lower extent (80). Also, using Peripheral Blood Mononuclear Cells (PBMC) activated with phytohemagglutinin (PHA), it has been shown that MSCs highly inhibit the proliferation of T_CM_, T_EM_, and effector CD4^+^ T cells (81). Moreover, Mareschi et al. observed that MSCs derived from different tissues such as bone marrow and placenta were able to decrease the proliferation of memory T cells (CD4^+^CD45RO^+^) (82). In particular, PBMC stimulated with PHA were shown to significantly decrease the frequency of CD4^+^ T_CM_ and T_EM_ cells, that produce TNF-α, IL-2, and IFNγ, when co-cultured with BM-MSCs (83).

Thereby, all these studies aiming at the evaluation of the inhibitory capacity of MSCs on human memory CD4^+^ T cells, demonstrate a stronger immunomodulatory effect on the T_CM_ cell subset. However, the effect exerted by MSCs on memory T cell subpopulations described to play a key role in RA immunopathogenesis, such as memory Th17 cells, memory Treg cells and memory Tfh cells among others still need to be investigated. Then will be describe the effect of MSCs on particular subpopulations memory T cells that could be related to the RA immunopathogenesis.

### Effects of MSCs on Effector Memory Vγ9Vδ2 T Cells

A high frequency of effector memory Vγ9Vδ2 T cells has been found in the peripheral blood and synovial fluid of RA patients. These cells have a potent capacity to secrete inflammatory factors, such as IFNγ and IL-17, and to present antigens (84). MSCs display a potent capacity to suppress the proliferation of γδ T cell, as well as their cytolytic responses and cytokine production (85, 86). This latter effect is mediated by the MSCs release of the COX-2-dependent production of prostaglandin E2 (PGE2) through their receptors, EP2 and EP4, expressed in Vγ9Vδ2 T cells (85, 86). These results suggest that MSCs exert a beneficial effect in RA through their capacity to prevent the immune response dysfunction mediated by γδ T cells via the inhibition of inflammatory cytokine production and the improvement of the anti-inflammatory response.

### Interaction Between Pro-inflammatory Memory Tfh Cells and MSCs

The production of auto-antibodies by B cells and thus the production of autoantibodies in RA patients involves in part the cooperation of Tfh cells (87). An association between an increased percentage of ICOS^+^ blood memory Tfh cells, auto-antibody titer of RA patient sera and the activity and/or severity of RA (88, 89). The differentiation of naïve CD4^+^T cells isolated from RA patients into Tfh cells was shown to be suppressed by human UC-MSCs in part through the indoleamine 2,3-dioxygenase (IDO) activity of MSC induced by IFNγ produced by Tfh cells (87). In the collagen-induced arthritis (CIA) model, MSC injection prevented arthritis progression in mice by altering both the number and function of Tfh cells (87). These results indicate that MSCs might inhibit the differentiation of Tfh toward the different memory subsets such as Tfh1, Tfh2, and Tfh17 and consequently decrease the auto-reactive B cell number and the production of auto-antibodies, such as anti-CCP.

### Effects of MSC on Pro-inflammatory Memory T Cells

Interactions between chemokines and their respective receptors are key mediators of inflammation since they govern the accumulation and homing of memory CD4^+^ T cells in the synovial membrane of RA patients. Chemokine ligand 3 (CCL3), CCL4, and CCL5 chemokines, which are highly produced by different cell types present in the synovial tissue, bind to various chemokine receptors such as CCR5 expressed at the surface of memory T cells that are (90, 91). CCR5 expression is increased at the surface of synovial tissue and fluid T cells and correlated with IFN-γ expression by synovial memory CD4^+^ T cells of RA patients (92–94). Synovial memory CD4^+^ T cells also express *lymphotoxin*-*alpha* (LT-α) that correlates with CCR6 expression and the presence of lymphocytic aggregates in synovial tissue (95). CCR6 was proposed to play a role in the development of aggregates of CD4^+^ T cells that are characteristically found in inflamed rheumatoid synovium (94).

As mentioned above, IL-17 plays a critical role in RA inflammatory process. IL-17 enhances the production of chemokines such as CCL20 and the stromal-derived factor 1 (SDF-1) by synoviocytes thus promoting the recruitment of memory T cells to the synovium (96–101). One of the mechanisms associated to the therapeutic effect of MSCs is their capacity to migrate and home into inflamed tissues (19). MSCs are well described to constitutively secrete a variety of different chemokines such as CCL2 (MCP-1), CCL3 (MIP-1α), CCL4 (MIP-1β), CCL5 (RANTES), CCL7 (MCP-3), CCL20 (MIP-3α), CCL26 (eotaxin-3), CXCL1 (GROα), CXCL2 (GROβ), CXCL5 (ENA-78), CXCL8 (IL-8), CXCL10 (IP-10), CXCL11 (i-TAC), CXCL12 (SDF-1), and CX3CL1 (fractalkine*)* (102–104). Furthermore, BM-MSCs express several chemokine receptors such as CXCR4, CCR1, CCR4, CCR7, CCR10, CCR9, CXCR5, and CXCR6 involved in MSCs migration (105). Thus, such MSCs could potentially migrate into the inflamed synovium and interact with memory T cells, inhibit their proliferation rate or/and alter their pro-inflammatory phenotype and finally reduce inflammation in the synovial membrane.

CXCR4 plays a central role in the homing and retention of CD4^+^ T cells (96, 106). Interestingly, RA patients with one or more susceptible HLA-DR haplotypes displayed a significantly higher frequency of memory CXCR4^+^CD4^+^ T cells, suggesting that synovial migration and retention of memory CXCR4^+^CD4^+^ T cells is associated with sustained auto-immunity and local inflammation. Moreover, the high frequency of memory CXCR4^+^CD4^+^ T cells correlated with the elevated expression level of HLA-DR on B cells underlying that B cells are important antigen-presenting cells in RA (107). Xie et al. have reported that MSCs exhibit an increased CXCR4 expression level when Notch signaling pathway was inhibited suggesting that notch signaling regulates MSC migration and function (108). Altogether these studies suggest that blocking of Notch pathway might enhance MSC therapeutic effect by increasing their capacity to migrate and home into the synovium where they will interact with memory CXCR4^+^CD4^+^ T cells and control RA pathogenesis.

### Effects of MSCs on Th17 and Treg Memory T Cells

Th17 cells express the retinoic acid-related orphan nuclear hormone receptor C (RORC) and secrete IL-17A along with other cytokines, including IL-17F, IL-21, and IL-22. Th17 cells are pro-inflammatory helper cells that protect the organism against extracellular pathogens, including Gram-negative bacteria, mycobacteria, and fungi (109). However, their deregulation is associated with the generation of auto-immune diseases including RA (109). On the other side, it is well known that human Treg cells play a central role in the maintenance of immune homeostasis and immunological self-tolerance (110). Treg cells exert potent immunosuppressive effects over effector T-cell proliferation and cytokine production through cytokine-independent mechanism requiring cell-to-cell contact. Treg cells are characterized by high expression level of CD25 (also referred as CD25 bright cells) and more specifically, intracellular expression of the transcription factor FoxP3 (111, 112). Moreover, Treg are characterized by a low expression of CD127 (IL-7 receptor alpha-chain) (113), and a down-regulation of CD127 which is associated with regulatory function acquisition (114). The imbalance between Th17 and Treg cells has been largely associated with the RA pathogenesis due to their close differentiation pathways but their completely opposite function. (115, 116). Indeed, Th17 cells are implicated in RA development and progression and high levels of IL-17 have been reported in the synovial fluid of RA patients which is positively correlated with the severity of the disease (117–120). Furthermore, IL-17 is mainly produced by CD4^+^CD45RO^+^ memory T cell (121, 122). Another molecule, the chemokine receptor CCR6, is expressed by memory Th17 cells and associated with their capacity to migrate toward inflammatory joints in response to CCL20 highly produced by T cells and synoviocytes (123, 124). On the other hand, CD4^+^CD25^high^ Treg cells are predominantly memory cells in the synovial fluid which is enriched with CD4^+^CD25^+^CD127l^°*w*^FoxP3^+^ Treg cells in the synovial fluid of RA patients (111, 125, 126). Furthermore, while the percentage of memory Treg cells subsets significantly increased in the synovial fluid of RA patients, it did not change in their peripheral blood, and this increased frequency of memory Treg correlated with the DAS28 (127). However, despite the increased number of Treg in the synovial fluid, inflammation is maintained suggesting an alteration of their functions in RA patients. This was confirmed by a body of studies that has demonstrated by the reduced regulatory functions of Treg derived from the peripheral blood (128–131) and the synovial fluid of RA patients (132). In line with these studies, Treg cells isolated from patients with active RA did not inhibit the secretion of pro-inflammatory cytokine such as IFN-γ and TNFα released by T effector cells (127–130, 133). Notoriously, TNFα can inhibit the suppressive function of Treg (129) suggesting that RA synovial fluid enriched in pro-inflammatory convert memory Treg cells into cells producing pro-inflammatory cytokines such as IL-17 unable to exert regulatory functions (134). An increased percentage of memory CD45RA^−^Foxp3^low^ non-regulatory T cells was reported in RA synovial fluid while it did not change in the peripheral blood of patients (55). Memory non-Treg cells produce IL-2, IFN-γ, and IL-17 and express high levels of *RORC* (135, 136).

MSCs are potent inhibitors of CD4^+^T-bet^+^CD183^+^ (Th1) and CD4^+^RORγt^+^CD161^+^ (Th17) cells proliferation and significantly reduce their capacity to produce pro-inflammatory cytokines such as IFN-γ, TNFα, and IL-1β (Th1) and IL-17A and IL-22 (Th17) (80). Indeed, using memory CD4^+^CD45RO^+^CCR6^+^ positive cells (Th17 cells), human BM-MSCs have been shown to induce the generation of Th17 cells with regulatory features in an inflammatory environment characterized by a decrease in *RORC* expression, an increase of FoxP3 expression and the acquisition of immunosuppressive functions (137).

Likewise, various studies have shown that MSCs have the capacity to increase the percentage of Treg cells *in vitro* in co-culture in mixed lymphocyte reactions (MLR) (138, 139). MSCs-derived PGE2 and transforming growth factor beta 1 (TGFβ1) are not redundant players in this mechanism (140). This was corroborated in a study with human adipose tissue-derived MSCs that were able to reduce IL-17, TNF, and IFN-γ production and to induce IL-10-producing T cells *in vitro* in collagen-specific peripheral blood T cells of RA patients (141). It is well admitted that MSCs co-cultured with purified CD4^+^ T cells induce the expression of CD25^High^ and FoxP3^+^ at the surface of these latter T cells in a contact-dependent manner (142, 143). The generation of these CD4^+^CD25^+^FoxP3^+^ Treg has been shown to be, in part, dependent on ICOSL expression by MSCs (142). Indeed, ICOS is expressed on activated memory T cells, including Th17 cells, thus through a contact cell-cell mechanism MSCs were proposed to interact with memory Th17 cells and generate memory Treg cells. In another study, it was reported that MSCs were able to recruit both CD4^+^CD25^+^CD45RA^+^ and CD4^+^CD25^+^CD45RO^+^ Treg cells, but the subpopulation of naïve Treg cells was recruited to a higher extent. Additionally, MSC regulate and maintain the suppressive function of memory Tregs cells over time (144). Therefore, in the context of RA, the regulation of memory Treg cell by MSCs is critical since they are more plastic than naive Treg cell population (136).

**Figure 1:**
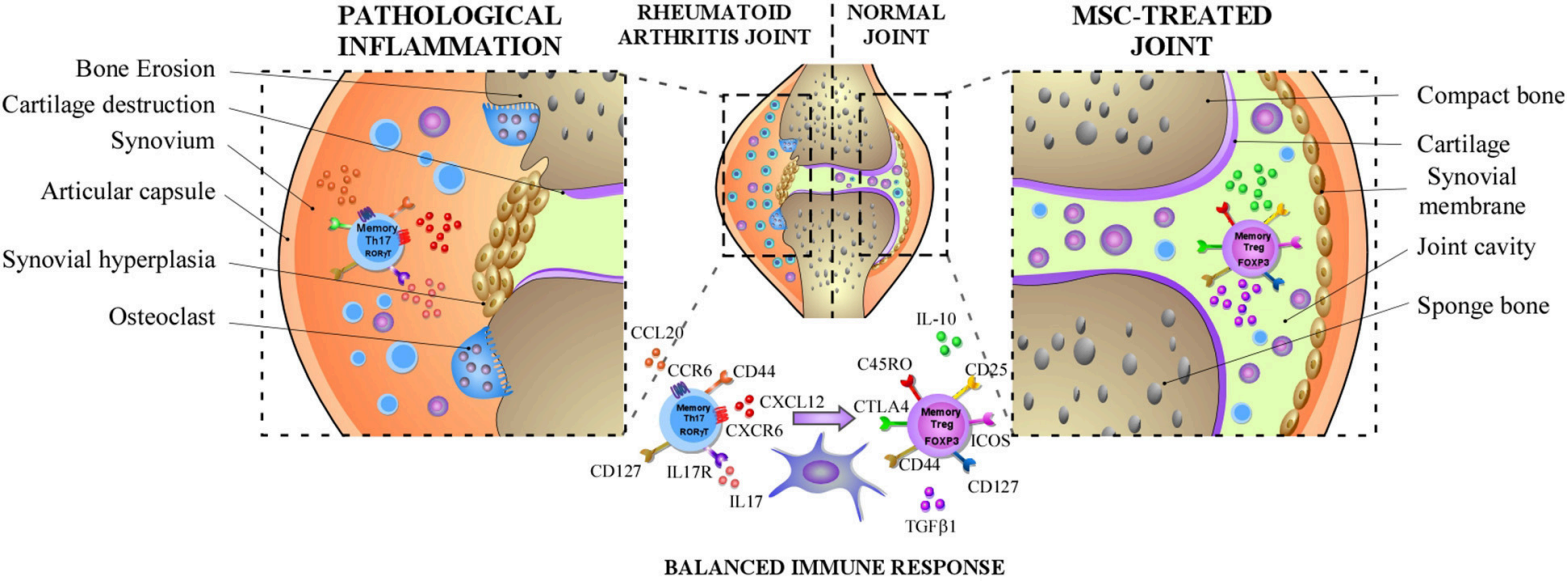
MSCs dampen RA progression through the induction of the balance between memory Th17 and Treg cells. In RA, MSCs can diminish the frequency of pathogenic memory Th17 cells and the production of pro-inflammatory cytokines such as IL-17, IL-22, and GM-CSF and promote their differentiation toward an anti-inflammatory phenotype. In parallel, MSCs might also increase the capacity of memory Treg cells to produce anti-inflammatory cytokines such as IL-10 or TGFβ1 and prolong their immunosuppressive capacity maintaining their anti-inflammatory phenotype.

Altogether, these studies provide evidence that MSCs do not only increase the generation of Treg cells and the production of IL-10 or TGFβ1 but also extend their immunosuppressive capacity maintaining their phenotype (FoxP3^+^ CD127^low^) and functions (140, 144). This is a critical function exerted by MSC, considering that Treg from RA patients exhibit an altered functionality. In addition, MSCs by suppressing the secretion of IL17-A by effector-memory Th17 cells decrease the acute or chronic activation of these cells in RA. Thus, MSCs do not only inhibit the IL-17 production but also induce the reprogramming of immunopathogenic memory Th17 cells toward T cells with regulatory phenotype and functions (137) (Summarized on Figure 1).

## Future Perspective

MSCs are multipotent cells with broad immunomodulatory properties, therefore, they have been proposed as the candidate of choice for autoimmune diseases treatment including RA. However, the clinical benefit for RA after 3 months of MSCs administration have shown inconsistent positive effects. Thus, it is necessary to increase the number of patients and studies in order to draw robust conclusions regarding MSC therapeutic effects in RA. Additionally, it is important to highlight that at today, clinical trials using MSCs were injected in patients with severe and refractory RA suggesting that MSCs treatment could be more effective at early stages of the disease (145). Also, the studies only evaluated the short-term efficacy of MSCs, from 3 to 8 months, and therefore the assessment of MSC long-term efficacy still needs to be addressed.

Based on the topics exposed here we believe that further studies needs to be address in order to evaluate the effect of MSC treatment on pathogenic memory T cells derived from RA patients. Since MSCs upon injection will migrate to the site of inflammation were they will find an elevated numbers of proinflammatory memory T cells it is essential to evaluated the effect of MSCs on RA memory T cells that has not been explored. Moreover, it is mandatory to achieve a detailed immune-monitoring of RA patients that analyses the dynamic of pathogenic and non-pathogenic memory T cells upon MSCs infusion.

## Conclusion

Memory T cells have been largely studied for their pivotal role in the pathogenesis of auto-immune disease such as RA. Although pro-inflammatory memory T cells-exhibit detrimental effect in RA, their potential plasticity offers an approach yet to be explored in order to better control RA progression. In this context, MSCs, potent immunosuppressive cells that are able to inhibit pro-inflammatory T cell proliferation and functions while inducing the generation of regulatory T cells, represent a strong candidate to choose for RA treatment. Thus, deciphering the basis of the crosstalk between MSCs and pathogenic memory T cells in RA will pave the way for developing novel and potent strategies to successfully improve MSC-based therapies.

## Author Contributions

NL-C, RC-L, FD, RE-V, and PL-C. wrote the manuscript with the input of MP-M, MT, SB, MW, and FE.

### Conflict of Interest Statement

The authors declare that the research was conducted in the absence of any commercial or financial relationships that could be construed as a potential conflict of interest.
